# Phylogenetic Position of a New *Trisetacus* Mite Species (Nalepellidae) Destroying Seeds of North American Junipers and New Hypotheses on Basal Divergence of Eriophyoidea

**DOI:** 10.3390/insects13020201

**Published:** 2022-02-15

**Authors:** Philipp E. Chetverikov, Brian G. Rector, Kirk Tonkel, Lindsay Dimitri, Denis S. Cheglakov, Anna E. Romanovich, James Amrine

**Affiliations:** 1 Zoological Institute, Russian Academy of Sciences, Universitetskaya Nab. 1, 199034 St. Petersburg, Russia; dcheglakov1@gmail.com; 2 Department of Invertebrate Zoology, St. Petersburg State University, Universitetskaya Nab. 7/9, 199034 St. Petersburg, Russia; aromanovich@gmail.com; 3 USDA-ARS-GBRRU, 920 Valley Road, Reno, NV 89512, USA; brian.rector@usda.gov (B.G.R.); kirk.tonkel@usda.gov (K.T.); lindsaydimitri@outlook.com (L.D.); 4 Division of Plant and Soil Sciences, West Virginia University, Morgantown, WV 26506, USA; james.amrine@mail.wvu.edu

**Keywords:** conifer pest, gall mites, host specificity, indel, seed ecology

## Abstract

**Simple Summary:**

Eriophyoid mites are microscopic herbivores associated with higher plants. Some of them are serious pests due to their ability to vector viruses and cause other damage to host plants. Mites of the genus *Trisetacus* are widespread parasites of conifers. They usually live in buds, cones, and rarely within needles of Pinaceae (pine family) and Cupressaceae (cypress family). We discovered a new species, *Trisetacus indelis* **n. sp.**, severely damaging seeds of three North American junipers in the western USA. This species possesses two morphologically different forms of females and has two deletion mutations in the gene cytochrome oxidase subunit I (*Cox1*). Such mutations are rare in eriophyoids and were previously detected only in two pestiferous species from palms and hazelnut. Our molecular-phylogenetic analyses determine the closest known relatives of the new species and suggest that Old and New World *Trisetacus* independently transitioned to living in seeds of junipers. Additionally we show that reconstruction of the phylogeny of Eriophyoidea based on one gene, *Cox1*, produces a poorly-resolved but biologically consistent tree topology to hypothesize the evolution of Eriophyoidea. Overall, our study improves our understanding of the diversity of conifer-inhabiting mites and indicates further needs in investigating the phylogeny of Eriophyoidea.

**Abstract:**

Eriophyoid mites of the genus *Trisetacus* Keifer are widespread parasites of conifers. A new oligophagous species, *T. indelis* **n. sp.**, was discovered severely damaging seeds of North American junipers (*Juniperus osteosperma*, *J. occidentalis*, and *J. californica*) in the western USA. It has two codon deletions in the mitochondrial gene *Cox1* rarely detected in Eriophyoidea and includes distinct morphological dimorphism of females. A phylogenetic analysis based on amino acid alignment of translated *Cox1* sequences using a large set of out-groups (a) determined that two North American congeners, *T. batonrougei* and *T. neoquadrisetus,* were the closest known relatives of *T. indelis* **n. sp.**, and (b) indicated that Old and New World seed-inhabiting *Trisetacus* from junipers do not form a distinct clade, suggesting a possible independent transition to living in seeds of junipers in America and Eurasia by *Trisetacus* spp. Our analysis produced a new topology consistent with a scenario assuming gradual reduction of prodorsal shield setation in Eriophyoidea and an ancient switch from gymnosperms to other hosts. Additionally, our analysis did not support monophyly of *Trisetacus*; recovered a new host-specific, moderately supported clade comprising *Trisetacus* and Nalepellinae (*Nalepella* + *Setoptus*) associated with Pinaceae; and questioned the monophyly of *Trisetacus* associated with Cupressaceae.

## 1. Introduction

Eriophyoid mites are an ancient group of phytoparasitic acariform mites exclusively associated with higher vascular plants and closely related to soil nematalycid mites [1,2,3]. Because of their microscopic size (about 200–300 μm), numerous undescribed species, and greatly simplified homogenous homoplastic morphology explained by miniaturization and adaptation to phytoparasitism, eriophyoids are among the most difficult groups of mites to classify taxonomically [4,5,6,7]. The identification of eriophyoids is usually based on laborious morphometrics and precise descriptions of morphology followed by a comparison with species descriptions scattered in different journals [8]. Since the beginning of the 21st century, DNA barcoding techniques typically complement morphological studies and have been proposed as a standard for taxonomic publications establishing new taxa in Eriophyoidea [9,10,11,12,13].

**Figure 1:**
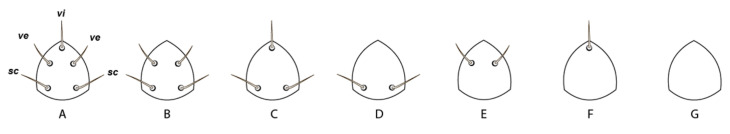
Prodorsal shield setation in large molecular clades of contemporary Eriophyoidea. (**A**)—*Pentasetacus* and *Loboquintus* (Pentasetacidae, sometimes called “five-setous” eriophyoids in literature [16,18,19]), (**B**,**E**)—Phytoptidae s.str., (**C**,**F**)—Nalepellidae, (**D**,**G**)—Eriophyidae s.l.

Currently accepted classification of Eriophyoidea assumes the presence of three families: Phytoptidae, Eriophyidae and Diptilomiopidae; differing in the number of prodorsal shield setae (*vi*, *ve*, and *sc*, Figure 1), presence or absence of opisthosomal setae *c1* and tibial solenidion *φI*, and morphology of the gnathosoma [4,14]. Molecular phylogenetic studies performed in the past decade [7,15,16] suggest a more complicated structure of Eriophyoidea resembling a combination of prior [17,18] and more modern [14] morphological classifications. They assume the presence of three large molecular clades, Nalepellidae restricted to coniferous hosts, Phytoptidae s.str. restricted to angiosperms, and Eriophyidae s.l. (Eriophyidae s.str. + Diptilomiopidae) inhabiting mainly angiosperms but also conifers and rarely ferns, plus two early-derived lineages from gymnosperms, *Pentasetacus* (from *Araucaria*) and *Loboquintus* (from *Cupressus*). Phylogenetic relations between these clades and the history of the colonization of terrestrial plants by eriophyoids remains poorly understood, however conifers were hypothesized as the earliest hosts [7,19,20].

**Figure 2:**
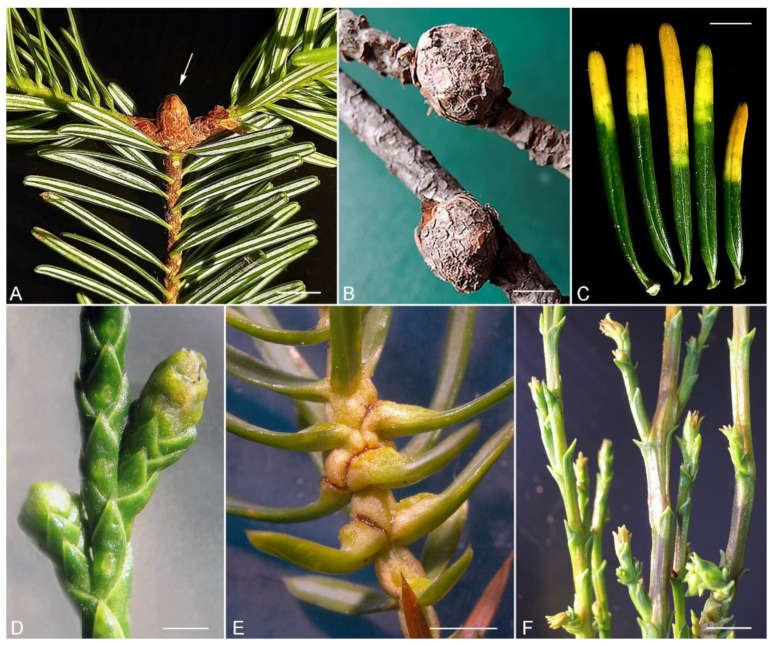
Damage caused by *Trisetacus* mites to conifers in the families Pinaceae (**A**–**C**) and Cupressaceae (**D**–**F**). (**A**)—bud of *Abies nordmanniana* (arrow) damaged by *Trisetacus* cf *bagdasariani* (Caucasus, Russia); (**B**)—bark galls on twigs of *Pinus sylvestris* caused by *Trisetacus pini* (Estonia); (**C**)—needles of *Abies nordmanniana* damaged by endoparasite *T. abietis* (Caucasus, Russia); (**D**)—male cone of *Juniperus virginiana* damaged by *Trisetacus juniperinus* s.l. (Crimea); (**E**)—swollen basal needles of *Juniperus communis* caused by *Trisetacus juniperinus* s.l. (northwestern Russia); (**F**)—deformed terminal buds of *Cupressus sempervirens* caused by *Trisetacus juniperinus* s.l. (Cyprus). Scale bars: (**A**) = 10 mm; (**B**–**F**) = 5 mm; (**D**) = 2 mm; (**E**) = 3 mm. Photos—P. E. Chetverikov.

Eriophyoid mites of the genus *Trisetacus* Keifer (Acariformes, Eriophyoidea, Nalepellidae) are common and widespread arthropod parasites of conifers [21,22]. They have been recorded in all regions where conifers are abundant except Australasia, where no studies on eriophyoids from gymnosperms have been performed to date [23]. All known hosts of *Trisetacus* belong to the two most diverse gymnosperm families, Pinaceae and Cupressaceae. Currently this genus comprises approximately 60 species; however, this number is expected to increase as more conifer species are investigated. Damage to host plants by *Trisetacus* can occur in a variety of forms [21,24]. Bud deformation (Figure 2A,F) is the most common damage caused by *Trisetacus* on various pinacean (*Abies*, *Picea*, *Larix*, *Cedrus*, *Pinus*, *Pseudotsuga*) and cupressacean (*Juniperus*, *Platycladus*, *Chamaecyparis*, *Cupressus*) host genera. Along with living in buds, several cryptic species of the complex *Trisetacus juniperinus* s.l., are also capable of infesting male cones and causing swollen galls on junipers (Figure 2D,E). Two endoparasitic species, *T. abietis* and *T. neoabietis* from *Abies* spp., live in air-cavities under the epidermis, causing necrosis of the parenchyma (Figure 2C) and premature needle fall [25,26]. A unique lineage of *Trisetacus*, *T. pini*, has adapted to live in galls, which they induce on young twigs on pines in Europe (Figure 2B).

**Figure 3:**
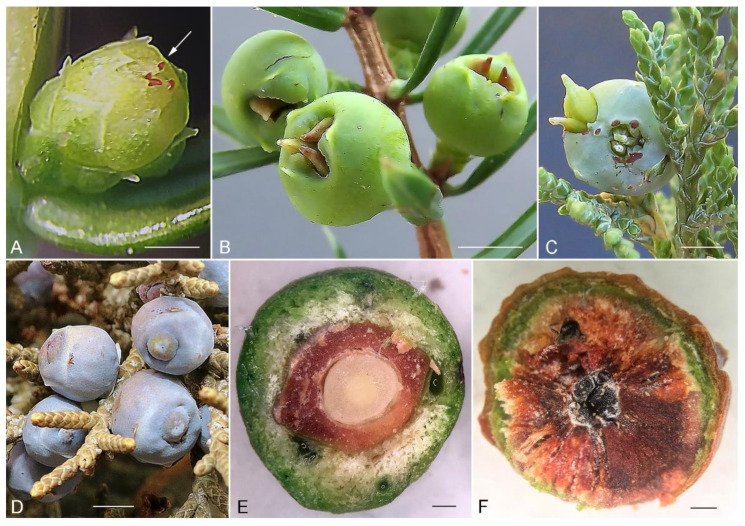
Damage caused by *Trisetacus* mites to cones and seeds of junipers. (**A**)—deutogyne females of *T. quadrisetus* (arrow) penetrating young female cone of *Juniperus communis* (Finland); (**B**–**D**)—enlarged seeds of *J. communis* (**B**), (Finland), *J. excelsa* (**C**), (Crimea), and *J. occidentalis* (**D**), (California, USA) protruding out of the female cones as a result of activity of *T. quadrisetus*, *T. kirghisorum*, and *T. indelis* **n. sp.**, respectively; (**E**)—dissected, undamaged *Juniperus californica* female cone; the outer green fruit with resin cells interspersed, along with the seed coat and central endosperm are clearly visible (California, USA); (**F**)—dissected *Juniperus californica* female cone occupied by *Trisetacus* mites; mite infestation results in the deformation of the developing embryo, rendering the seed inviable by converting the endosperm into layered, spongy tissue (California, USA). Scale bars: (**A**,**E**,**F**) = 1 mm; (**B**) = 4 mm; (**C**) = 3 mm; (**D**) = 2 mm. Photos—P. E. Chetverikov (**A**–**C**), L. Dimitri (**D**), and K. Tonkel (**E**,**F**).

Damaging female cones and living inside seeds is typical only for *Trisetacus* associated with junipers (Figure 3). Along with bud mites, e.g., *T. juniperinus* severely damaging cypress trees in Europe [27], seed mites are serious pests of junipers on different continents. *Trisetacus kirghisorum* causes serious problems in central Asia where it heavily damages cones and seeds and notably slows growth and reproduction of economically and culturally important *Juniperus* spp. [28,29]. Similarly a common European mite species, *T. quadrisetus*, and the North American species *T. batonrougei* and *T. neoquadrisetus* can destroy up to 95% of seeds of their juniper hosts [30]; (J. Amrine and P. Chetverikov, unpublished observations). This group of seed parasites is a potential serious threat to conifer nurseries and requires early detection and removal of infected plants.

During field surveys in mountainous areas of the western USA we observed groves of junipers heavily infested by a new *Trisetacus* species. In this paper we aim to describe this species and assess its phylogenetic position within the genus *Trisetacus*, based on sequence comparisons of the mitochondrial cytochrome oxidase c gene (*Cox1*), including a dataset from a previous study [23]. In addition, we investigated the phylogenetic structure of Eriophyoidea using a new set of outgroups and a translated *Cox1* amino-acid alignment that was twice as long as that produced by the classical LCO-HCO fragment [31].

## 2. Materials and Methods

**Morphology**. Live mites of *T. indelis* **n. sp.** were collected in 2021 in the USA from plants using a fine minuten pin and a dissecting microscope, then placed in Eppendorf tubes filled with 96% ethanol. The mites were mounted in modified Berlese medium with iodine [32] and cleared on a heating block at 90 °C for 3–5 h. Slide-mounted specimens were examined with differential interference contrast light microscopy (DIC LM) using a Leica DM2500 (Leica Microsystems GmbH, Wetzlar, Germany) and photographed with a ToupCam UCMOS09000KPB digital camera (Hangzhou ToupTek Photonics Co., Hangzhou 310030, China). Images and specimens were analyzed and measured using ToupTek ToupView software (Hangzhou ToupTek Photonics Co., Hangzhou 310030, China). In the description of the new species, measurements of a holotype (female) are presented followed by measurement ranges for paratype females. All measurements are given in micrometers (μm) except when specified otherwise. Classification and terminology of morphology follow [4,14,33]. The scientific names of host plants are given according to [34]. Drawings of mites were sketched by pencil using a video projector [35], scanned, and finalized in Adobe Illustrator CC 2014 (Adobe Systems, San Jose, CA, USA) using a Wacom Intuos S СTL-4100K-N (Wacom Co., Ltd, Kazo, Saitama, Japan) graphics tablet.

**DNA extraction and sequencing**. For DNA extraction, 1–3 mite specimens of each species were crushed with a fine pin in a 3 μL drop of distilled water on a cavity well microscope slide. The drop was pipetted into a thin-walled PCR tube with 30 μL of 5% solution of Chelex**^®^** 100 Resin (Bio-Rad Laboratories, Inc., Hercules, California, USA) before being heated three times (5 min at 95 °C). The solution above the settled Chelex**^®^** granules was used as the DNA template for PCR to amplify a fragment of subunit I of *Cox1*. Thermal cycling profiles and primers (for PCR and for sequencing) used were as specified by [36]. After amplification, 4 μL of each reaction product was mixed with 0.5 μL of SYBR Green I (Lumiprobe, Hannover, Germany) and analyzed by electrophoresis in a 1% agarose gel to assess the product size and concentration. Sequences were obtained using BigDye Terminator v.3.1 chemistry in a 3500xl Genetic Analyzer (Applied Biosystems, Foster City, CA, USA). Trace files were checked and edited using GeneStudioTM Professional 2.2.0.0. (www.genestudio.com, accessed on 10 February 2022).

**Sequence alignment and molecular phylogenetic analyses**. A large set of closely related and distant eriophyoid out-groups sequenced in previous studies were included in the analysis: complete dataset of Nalepellidae [23], all *Cox1* gene sequences of Eriophyidae s.l. from Genbank that are longer than 1100 bp, ten sequences of phytoptids, and three sequences of pentasetacids. The tree was rooted with sequences of *Nanorchestes* KY922356.1 and *Gordialycus* KY922376.1 [3]. Sequences were aligned using the L-INS-i MAFFT algorithm [37] and the web-based program interface [38] with the default settings (scoring matrix 200PAM/k = 2, gap opening penalty = 1.53, offset value = 0.0). The absence of stop codons in *Cox1* sequences was checked with Mega X [39]. The final alignment contained 131 sequences with 1158 nucleotide positions. The *Cox1* sequences were translated into amino acid sequences and maximum likelihood analysis was conducted in IQ-tree 2 [40]. For amino-acid evolution, mtART+R4 model was selected using ModelFinder [41] as implemented in IQ-tree 2 based on the Bayesian Information Criterion. Branch support values were generated from Ultrafast bootstrap approximation (UFBoot) with 10,000 bootstrap alignments, 1000 maximum iterations and a minimum correlation coefficient of 0.99 [40].

## 3. Results

### 3.1. Morphological Description of Trisetacus indelis **n. sp.**

**Figure 4:**
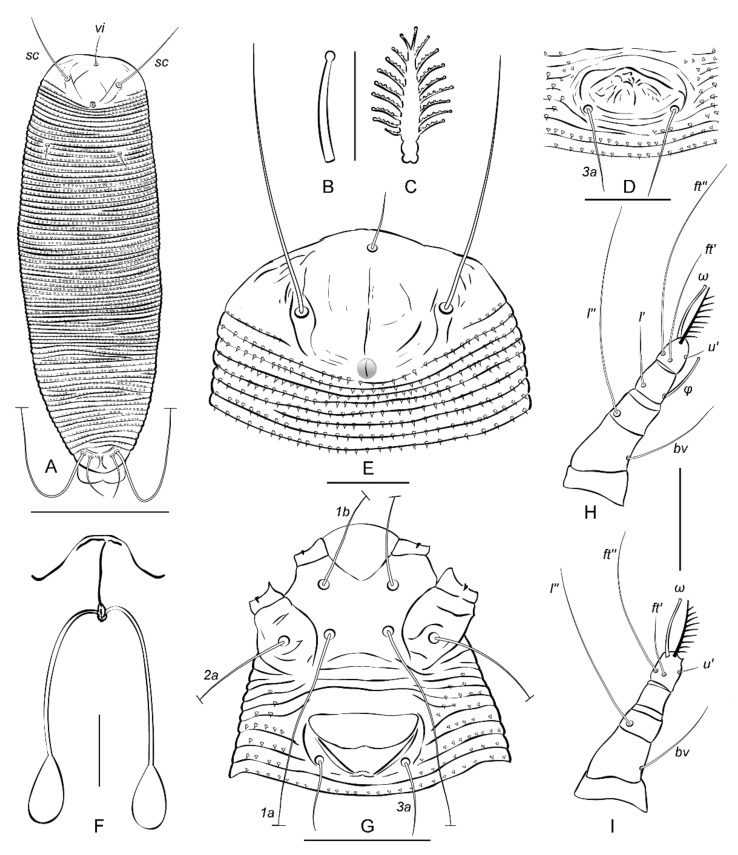
Drawings of *Trisetacus indelis* **n. sp.** (protogyne form). (**A**)—dorsal view of entire mite, (**B**)—tarsal solenidion I, (**C**)—empodium (**D**,**I**)—male genital area, (**E**)—prodorsal shield, (**F**)—female internal genitalia, (**G**)—coxigenital area, (**H**)—leg I, (**I**)—leg II. Scale bars: (**A**) = 70 μm; (**C**,**D**) = 10 μm; (**E**,**F**,**H**,**I**) = 15 μm; (**D**) = 20 μm; (**G**) = 30 μm.

**Figure 5:**
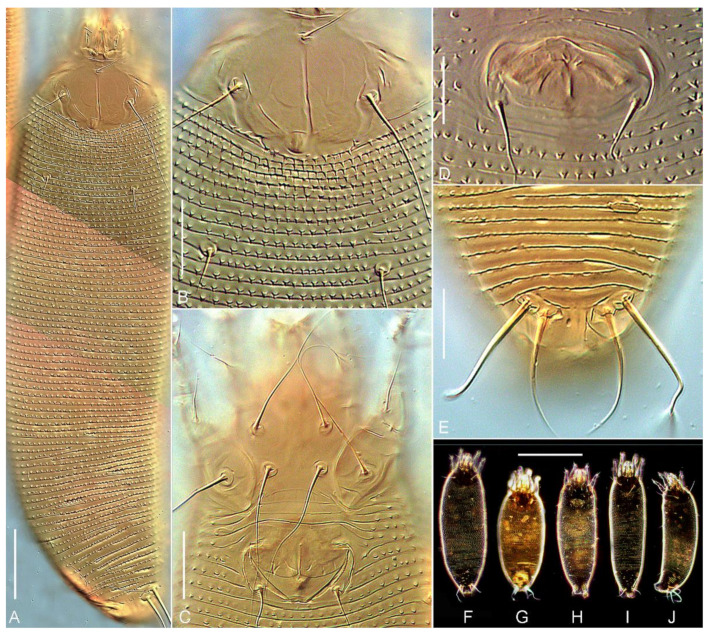
DIC LM (**A**–**E**) and pseudo dark-field (**F**–**J**) microphotographs of protogynes (**A**–**H**) and deutogynes (**I**,**J**) of *Trisetacus indelis* **n. sp.** (**A**)—dorsal view of whole female, (**B**)—female prodorsal shield, (**C**)—female coxigenital area, (**D**)—male genital area, (**E**)—dorsal view of telosoma, (**F**)—typical slide-mounted protogyne female under lower magnification, (**G**)—collapsed protogyne female with constricted opisthosomal muscles, (**H**)—typical slide-mounted male, (**I**)—dorsal view of deutogyne female, (**J**)—lateral view of deutogyne female. Scale bars: A = 30 μm; (**B**,**C**,**E**) = 15 μm; (**D**) = 10 μm; (**F**–**J**) = 100 μm.

**Figure 6:**
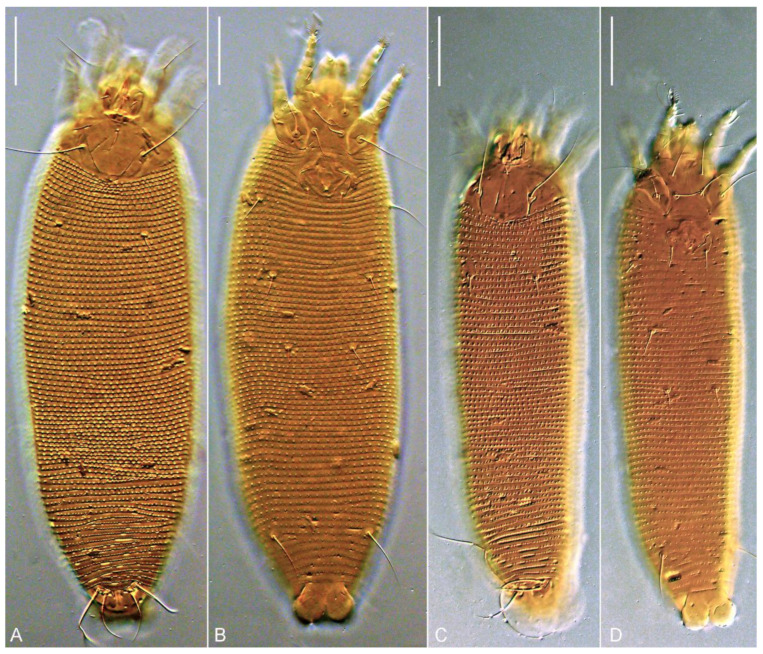
DIC LM images showing typical size differences between protogyne (**A**,**B**) and deutogyne (**C**,**D**) females of *Trisetacus indelis* **n. sp.** (**A**,**C**)—dorsal view, (**B**,**D**)—ventral view. Scale bar = 30 μm.

**PROTOGYNE FEMALE (n = 10,**Figure 4, Figure 5 and Figure 6**),** Body vermiform, white and slightly yellowish color 267 (252–278), 69 (63–72) wide at the level of setae *c2*. **Prodorsal shield** subrhomboidal or subcordial, 29 (28–33), 51 (48–55) wide, without frontal lobe. Prodorsal shield ornamentation variable, usually comprised of median, two submedian I, two submedian II, and several additional short lines. Median line appears concave, projecting from posterior margin of prodorsal shield up to the base of tubercle *vi*; in some females median line can be fragmented, very faint and almost imperceptible. Admedian lines absent like in other *Trisetacus* spp., associated with Cupressaceae [23,30]. Submedian I short, ridge-like, can be broken and fragmented; right and left submedian I converge postero-medially forming in most studied mites a figure resembling a flattened U. In some mites submedian I is forked anteriorly with a short internal branch going obliquely towards tubercle of *vi* (Figure 4A). Submedian II present only outside tubercle of *sc*, usually consists of a series of short fragments. Epicoxal area with short and sometimes curved lines. No microtubercles or microgranulations on prodorsal shield and epicoxal area. A subcuticular globose apodeme, typical for most *Trisetacus* from Cupressaceae, present near posterior margin of prodorsal shield. Prodorsal shield setae: *vi* 12 (10–14), directed up and anteriad; *sc* 51 (46–57), 26 (24–27) apart, directed up and anterolaterad; distance between tubercles of *vi* and *sc* 17 (16–18). **Gnathosoma** directed obliquely down and forward; palps 25 (24–26). Gnathosomal setae: seta *ν* 0.5 (0.5–1); pedipalp genual seta *d* non-bifurcate, 9 (8–10); pedipalp coxal seta *ep* 3 (3–4). Suboral plate rounded anteriorly, smooth.

**Leg I** 27 (25–30), tarsus 6 (4–6), *u’* 3 (2–3)*, ft’* 13 (11–16), *ft’’* 25 (21–28), *ω* 9 (9–10) with small spherical knob; empodium 9 (8–10), 9/9-rayed, all rays except terminal pair with 4–6 tiny processes each; tibia 5 (4–6), *l’* 6 (3–6); *φ* 7 (7–9); genu 5 (5–6), *l’’* 26 (22–30); femur 8 (8–10), *bv* 14 (12–15). **Leg II** 24 (23–26), tarsus 5 (4–5), *u’* 3 (2–3)*, ft’* 8 (7–12), *ft’’* 22 (21–27), *ω* 9–10 with small spherical knob; empodium 9 (8–10), similar to empodium I; tibia 5 (4–5); genu 5 (4–5), *l’’* 22 (21–28); femur 7 (7–9), *bv* 12 (11–15).

**Coxal plates I** smooth, coxal plates **II** with several short, curved ridges near tubercles of *2a*; coxal setae *1b* 26 (27–34), 16 (15–16) apart; *1a* 37(34–42), 12 (12–14) apart; *2a* 52 (50–61), 31 (30–33) apart; all coxal setae are very thin in distal half and break off easily during specimen preparation. Prosternal apodeme inconspicuous; cuticle between tubercles of coxal setae *1a* smooth; 2 (2–3) incomplete and 1 complete coxigenital annuli before epigynium. **External genitalia.** Genital coverflap distally rounded, smooth, with medioposterior indentation; 7 (7–8) long, 27 (26–28) wide; setae *3a* 18 (16–22), 17 (17–19) apart. **Internal genitalia (n = 4).** Spermathecae tear-drop shaped, 12–15 long, 7–9 wide; spermathecal tubes narrow, non-recurved, 30–35 long, 1.5–2 wide without widening; spermathecal process absent; longitudinal bridge 12–15; anterior genital apodeme trapezoidal, distinct; oblique apodeme absent.

**Opisthosoma** dorsally with 76 (73–79) annuli, ventrally with 71 (68–76) annuli between posterior margin of coxae II and caudal lobes. Setal lengths: *c1* 8 (8–11), *c2* 45 (40–48), *d* 37 (32–43), *e* 15 (11–16), *f* 40 (38–44); *h1* 25 (22–27); *h2* 112 (100–122); 10 (10–11) annuli from rear shield margin to *c1*; 10 (7–10) annuli from rear shield margin to *c2*; 12 (11–13) annuli between *c2*–*d*; 13 (12–16) annuli between *d* and *e*; 31 (30–34) annuli between *e* and *f*; 5 (5–6) annuli between *f* and *h2*.

**MALE (n = 4).** Body vermiform, 214–266, 55–59 wide, white or slightly yellowish. Prodorsal shield 28–32, 49–52 wide. Leg I 27–29, leg II 25–26, empodium 8/8- or 7/8-rayed. Ornamentation of prodorsal shield similar to that of females. Genital area 11–12 long × 24–25 wide, setae *3a* 16–22, 20–21 apart.

**DEUTOGYNE FEMALE (n = 10).** Difference in color is the only qualitative trait distinguishing deutogynes and protogenes. Deutogynes are reddish. Slide-mounted deutogynes are notably shorter and narrower than protogynes (Figure 5); however, if protogynes are collapsed (perhaps due to opisthosomal muscle contraction during heating the slide), the difference in length may be masked (e.g., Figure 5G vs. Figure 5I). Deutogynes are about 10% shorter (186–223 vs. 252–278) and 10% narrower (51–57 vs. 63–72) than protogynes, have fewer dorsal (61–67 vs. 73–79) and ventral (59–65 vs. 68–76) annuli, narrower genital coverflap (19–23 vs. 26–28), and longer setae *e* (27–36 vs. 11–16). Additionally, most of the deutogynes possess fewer empodial rays on legs I and II (8/8 *vs* 9/9), although some transitional forms of deutogynes with 8/9- and 9/9-rayed empodia were also observed.

**Type material.** Holotype female from slide E4679, paratype females and males from slide series E4680–E4683 collected on 24 February 2021 near Madeline, California (41.129000, −120.501000), from inside enlarged seeds of *Juniperus occidentalis* Hook. (Cupressaceae). Type material is deposited in the Acarological Collection of the Zoological Institute of the Russian Academy of Science (ZIN RAS) in Saint-Petersburg and in the collection of eriophyoid mites of J. Amrine (West Virginia University, USA).

**Relation to host.** Mites penetrate immature female cones, feed on seed endopsperm, and cause seed deformation; infested seeds may protrude from the cones up to 1–2 mm (Figure 3D–F).

**Additional material.** Females and males of *Trisetacus indelis* **n. sp.** from slide series E4684–E4688 collected on 10 February 2021 in the Sweetwater Range, Nevada (38.607000, −119.240000) from inside enlarged seeds of *Juniperus osteosperma* Hook.; and from slide series E4689–E4693 collected on 9 February 2021, near Dales, California (40.291000, −122.112111) from inside enlarged seeds of *J. californica* Carr. (Cupressaceae). This material has been deposited in the Acarological Collection of ZIN RAS, Saint-Petersburg, Russia.

**Etymology.** The specific epithet, *indelis*, is an adjective, gender masculine, derived from “indel” and corresponding to the codon deletion mutations present in the *Cox1* sequence of this species in comparison to all currently known members of the genus *Trisetacus* [23].

**Differential diagnosis.***T. indelis* **n. sp.**, is morphologically close to *T. kirghisorum* and *T. batonrougei*. Main differences between them concerns prodorsal shield ornamentation and shapes of the spermathecal apparatus. In *T. batonrougei* median and admedian lines are absent [30], whereas in *T. kirghisorum* three short lines (median and two admedian) [42] and in *T. indelis* **n. sp.** one long median line are present (Figure 4E; Figure 5A,B). In *T. indelis* **n. sp.** the spermatheca is small, tear drop-shaped, and the spermathecal tube is long and narrow (Figure 3F). In *T. kirghisorum* and *T. batonrougei* the spermatheca is notably larger and cucumber-shaped, and the spermathecal tube in *T. batonrougei* has an additional expanded segment [23].

### 3.2. GenBank Data and Cox1 Sequence Diversity

Seven nearly identical *Cox1* sequences of *T. indelis* **n. sp.** were obtained (K2P distances = 0.000–0.001, Table 1). Two sequences (isolates ost6 and ost10) have one synonymous substitution (C/Y vs T in all other sequences) in the position 852 of our alignment in a codon corresponding to asparagine. All sequences of *T. indelis* **n. sp.** are lacking two nucleotide triplets (alignment positions 484–489) in comparison to all other known *Trisetacus* spp. [23]. Among all other (approximately 1500) *Cox1* sequences of eriophyoid mites that are currently present in GenBank, codon deletions are present only in the 21 sequences of *Phytoptus avellanae* (1 codon deletion) and four sequences of *Retracrus* (2 codon deletions). They are situated in the same region of the *Cox1* gene (alignment positions 469–474) as in *T. indelis* **n. sp.**

### 3.3. Molecular Phylogenetic Analysis 

**Figure 7:**
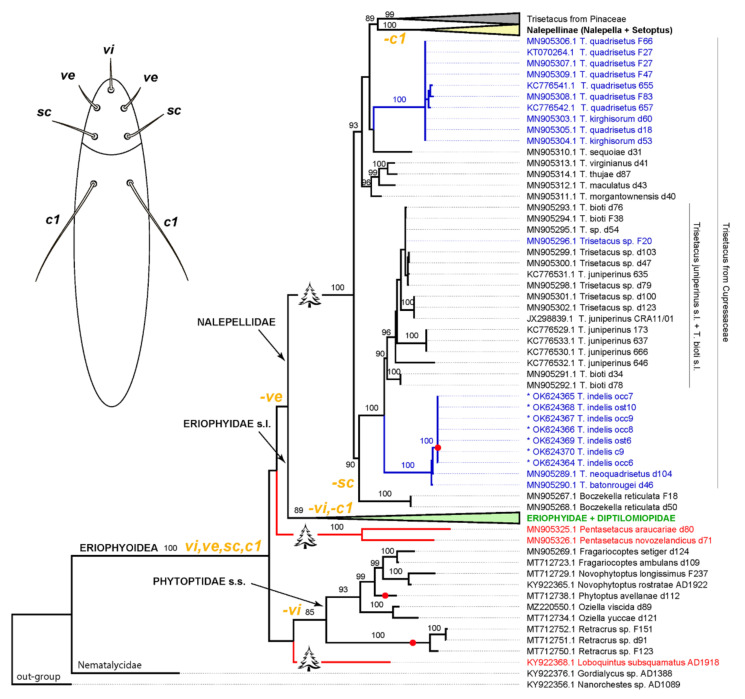
Maximum likelihood tree based on a 386-amino-acid alignment of the translated *Cox1* gene showing phylogeny of Eriophyoidea and position of *Trisetacus indelis* **n. sp.** (asterisks). Nodes are labeled by UF bootstrap support (UFBS), only UFBS ≥ 85 are shown. Conifer pictograms indicate lineages exclusively associated with gymnosperms. Collapsed lineages are: *Trisetacus* from Pinaceae (gray), Nalepellinae (yellow), and Eriophyidae s.l. (green). Clades comprising five-setous pentasetacids are colored red and those comprising *Trisetacus* from seeds of junipers are colored blue. Red spots indicate eriophyoid lineages with codon-deletion mutations in the *Cox1* gene. The diagram above left shows the position of paired setae *vi*, *ve*, *sc*, and *sc* in a hypothetical pentasetacid-like ancestor of Eriophyoidea. The hypothetical loss of these setae marking major eriophyoid lineages is indicated near corresponding branches (orange).

The molecular phylogenetic analysis revealed a monophyletic Eriophyoidea, as expected, comprising three large clades (Phytoptidae s.str., Eriophyidae s.l, and Nalepellidae) with poorly resolved relations between them (Figure 7). Two archaic pentasetacid genera (*Loboquintus* and *Pentasetacus*) were situated in the unresolved basal part of the tree and clustered with different large eriophyoid clades. The genus *Trisetacus* was polyphyletic. All members of *Trisetacus* associated with Pinaceae formed a well-supported clade sister to Nalepellinae (*Setoptus* + *Nalepella*), which are also associated with Pinaceae. *Trisetacus* from Cupressaceae appeared as polyphyletic and comprised several well-supported clades, each of them including mite species associated with different species of the plant genus *Juniperus*. The mite genus *Boczekella* was sister to one of the clades of *Trisetacus* from Cupressaceae. *Trisetacus* spp., living in seeds of *Juniperus* spp., did not cluster together. However, all analyzed species (*T. batonrougei*, *T. quadrisetus*, and *T. indelis* **n. sp.**) from seeds of American junipers (*Juniperus californica*, *J. occidentalis*, *J. osteosperma*, and *J. virginiana*) formed a highly supported clade sister to a clade comprising a complex of cryptic species, viz. *Trisetacus juniperinus* s.l. + *T. bioti* s.l. associated with various cupressacean genera (*Cupressus*, *Juniperus*, *Platycladus*). Only one population from this complex (*Trisetacus* sp. F20 from Crimea) lives in seeds. The species *T. quadrisetus*, *T. kirghisorum*, and *Trisetacus* sp. F20 associated with seeds of the Eurasian junipers *J. excelsa*, *J. foetidissima*, and *J. communis*, were not clustered together although monophyly of the lineage *T. quadrisetus* + *T. kirghisorum* was strongly supported. 

## 4. Discussion

**A new mite species destroying seeds of North American junipers.** In this study, we report on a new *Trisetacus* species, *T. indelis* **n. sp.**, associated with junipers native to the western USA. This species is remarkable in several aspects. First, this is the first member of Nalepellidae possessing codon-deletion mutations in the *Cox1* gene. Such mutations in *Cox1* are rare in Eriophyoidea, currently known in only two other eriophyoid genera (both from Phytoptidae s.str.): *Phytoptus* and *Retracrus* [36,43]. In all cases, the codon deletions occur in approximately the same region, about 500 bp in the 3**′** direction from the *Cox1* start codon, which should be noted in future when designing primers.

Second, the new species is a new distinct example of deuterogeny, with two morphologically different forms of females present in the life cycle that possess identical *Cox1* barcodes [44,45]. Among other members of *Trisetacus*, distinct deuterogeny has been reported for *T. kirghisorum* and *T. piceae*. However, contrary to all known examples of deuterogeny in Eriophyoidea, in these latter two species both sexes (females and males) are subjected to seasonal dimorphism [29,46,47]. This fact possibly correlates with the closer relation of *T. kirghisorum* to the clade comprising *Trisetacus* from Pinaceae (which includes *T. piceae*) than to the clade comprising *Trisetacus* from seeds of American junipers revealed in the molecular phylogenetic analysis presented here (Figure 7). Although a deep investigation of the problem of deuterogeny is out of the scope of this study, we hypothesize that deuterogeny may be common for many *Trisetacus* spp., and that possibly all eriophyoids may have seasonally adapted female forms that differ in their morphologies and physiological functions to varying extents in different phylogenetic lineages.

Third, *Trisetacus indelis* **n. sp.**, provides a new documented case of oligophagy in Eriophyoidea, a superfamily in which most members are considered highly host-specific monophages [48]. This species inhabits seeds of three partially sympatric species of *Juniperus* (*J. californica*, *J. occidentalis*, and *J. osteosperma*) growing in mountainous areas of the western USA [49]. Our analyses suggest that this mite species is closely related to the two other presumed monophagous North American *Trisetacus* species, *T. batonrougei* and *T. quadrisetus,* associated with *Juniperus scopulorum* and *J. virginiana*, respectively, which have a more eastern distribution in USA than the known hosts of *T. indelis* **n. sp.** Because *Cox1* is a relatively quickly evolving gene often used for delimiting host races and cryptic species [50,51,52,53,54,55], we expected to see variation, particularly in 3d codon positions, between populations of *T. indelis* **n. sp.**, from different hosts and localities. The observed striking pairwise sequence similarity and presence of the indicative indels, which are very rare in *Cox1* genes of Eriophyoidea, suggest that *T. indelis* n. sp., may be a recently evolved species, quickly expanding its host range and area of distribution. The single synonymous nucleotide substitution observed in a population of *J. osteosperma* (isolates ost6 and ost 10) may be a signature of early host-dependent species divergence in the form of a point mutation. Future studies are needed to trace the evolutionary history of *T. indelis* **n. sp.**, and assess its impact on the seed ecology of its hosts, two of which (*J. occidentalis* and *J. osteosperma*) are considered weedy in parts of their native ranges [56].

**Implications to the phylogeny of Eriophyoidea.** No morphological or multigene molecular phylogenetic analyses to date have resolved the basal phylogeny of Eriophyoidea [3,6,7,15,16,36,57]. One general conclusion from prior studies was that genes usually used in molecular phylogenetics by acarologists (rDNA, *Cox1*, 16S) are suboptimal for resolving the phylogeny of Eriophyoidea. However, it was noted that in many cases when it was possible to obtain well-resolved multigene phylogenies of different acarine groups, it has often largely reproduced the topology of trees based only on the *Cox1* gene [58]. Our analyses based on translated *Cox1* amino acid alignments produced an incompletely resolved tree of Eriophyoidea but revealed several new groupings marked by host associations and common morphology.

The analysis presented here unambiguously placed *Trisetacus indelis* **n. sp.**, within a clade of American seed-infesting mites from junipers. Similar to a previous phylogeny of Nalepellidae based on the D1D2 28S fragment [23], our current analysis (a) questions the monophyly of *Trisetacus*, (b) produces a new moderately supported clade from pinacean hosts (Nalepellinae + *Trisetacus* from Pinaceae), (c) rejects monophyly of *Trisetacus* from Cupressaceae, (d) suggests a possible independent transition of *Trisetacus* to living in seeds of New and Old World junipers, and (e) again indicates an uncertain position of *Boczekella* associated with Laricoideae (Pinaceae) in the basal part of the nalepellid clade. Additionally, our analysis offers a new hypothesis on the basal divergence of Eriophyoidea suggesting (i) gradual loss of prodorsal shield setae (*vi*, *ve*, *sc*), and opisthosomal setae c1 in major eriophyoid lineages—Nalepellidae, Phytoptidae s.str., and Eriophyidae s.l., (ii) gymnosperms as the most likely ancestral hosts of Eriophyoidea, and (iii) pentasetacids as an archaic polyphyletic group comprising “living fossils.” While these hypotheses have been widely discussed in the relevant literature [7,16,18,19,20,36,59,60], the presented topology is the first case in which all of them are combined together. Future analyses based on larger mitogenomic and genomic datasets may produce a breakthrough in our knowledge of the early steps of the evolution of Eriophyoidea and generate a phylogenetically consistent classification of this important taxon.

## Figures and Tables

**Table 1:** Characteristics of the *Cox1* sequences of *T. indelis* **n. sp.** obtained in this study.

GB Accession Number	Isolate	Length	Female Form	Host Species
OK624364	occ6	696 bp	deutogyne	*Juniperus occidentalis*
OK624365	occ7	1152 bp	protogyne	*Juniperus occidentalis*
OK624366	occ8	1152 bp	deutogyne	*Juniperus occidentalis*
OK624367	occ9	1152 bp	protogyne	*Juniperus occidentalis*
OK624368	ost10	1152 bp	deutogyne	*Juniperus osteosperma*
OK624369	ost6	1103 bp	protogyne	*Juniperus osteosperma*
OK624370	c9	632 bp	protogyne	*Juniperus californica*

## Data Availability

Microscopy slides: type, paratype, and additional material is deposited in the Acarological Collection of the Zoological Institute of the Russian Academy of Science (ZIN RAS) in Saint-Petersburg and in the collection of eriophyoid mites of J.A. (West Virginia University, USA). DNA sequences: Genbank accessions OK624364-OK624370 (https://www.ncbi.nlm.nih.gov/nuccore/OK624364, accessed on 10 February 2022). The alignment analyzed in this study is available on request from the corresponding author.
